# Septic Cardiomyopathy: Age-Dependent Physiology and Hemodynamic Aspects—A Narrative Review

**DOI:** 10.3390/children13020239

**Published:** 2026-02-08

**Authors:** Marianna Miliaraki, George Briassoulis, Evangelia Dardamani, Panagiotis Briassoulis, Stavroula Ilia

**Affiliations:** 1 Division of Pediatric Intensive Care, School of Medicine, University of Crete, 71300 Heraklion, Greecestavroula.ilia@uoc.gr (S.I.); 2 Postgraduate Program “Emergency and Intensive Care in Children, Adolescents and Young Adults”, School of Medicine, University of Crete, 71300 Heraklion, Greece; briasoug@uoc.gr; 3 Division of Anesthesiology, School of Medicine, National and Kapodistrian University of Athens, 12462 Athens, Greece; briaspan@med.uoa.gr

**Keywords:** adult, pediatric sepsis, septic shock, sepsis-induced cardiomyopathy, hemodynamic monitoring, cardiac output

## Abstract

**Highlights:**

**What are the main findings?**

- Sepsis-induced cardiomyopathy (SCM) is an acute, reversible, non-ischemic myocardial dysfunction in children and adults, ranging from subclinical biventricular impairment to overt cardiogenic shock.
- Pediatric SCM often presents with hypodynamic profiles, while adults frequently show hyperdynamic, vasoplegic states, reflecting age-dependent differences in cardiovascular physiology.
- Multimodal diagnostic approaches, including echocardiography and advanced hemodynamic monitoring, improve detection of subtle or evolving myocardial dysfunction.

**What is the implication of the main findings?**

- SCM requires repeated, physiology-driven, multi-modal cardiovascular assessment to guide individualized management.
- Understanding age-specific hemodynamic profiles can help tailor interventions and improve outcomes in both adult and pediatric patients.

**Abstract:**

Background: Septic cardiomyopathy (SCM) is a dynamic and heterogeneous complication of sepsis, driven by systemic inflammation, autonomic dysregulation, and microcirculatory alterations. Pediatric and adult patients share common pathophysiologic mechanisms, but age-dependent differences in cardiovascular physiology produce distinct hemodynamic responses. Methods: A structured narrative review of clinical and experimental studies published between 2000 and 2025 was conducted via PubMed and major critical care literature. Studies were included if they addressed SCM pathophysiology, hemodynamic monitoring, and therapeutic strategies across age groups, while studies focusing on non-septic cardiac dysfunction were excluded. Results: Adult SCM often presents as hyperdynamic, vasoplegic states, whereas pediatric patients more frequently exhibit hypodynamic profiles, reflecting developmental differences in myocardial reserve and autonomic regulation. Evidence suggests that isolated conventional echocardiographic parameters may underestimate myocardial impairment, whereas advanced modalities, including myocardial strain echocardiography and multimodal hemodynamic monitoring, may serve as complementary tools to detect subtle or evolving myocardial dysfunction. Pediatric evidence remains limited, and therapeutic guidance is largely extrapolated from adult studies. Conclusions: SCM should be approached as a time-dependent, physiology-driven condition, requiring repeated, integrated multimodal cardiovascular assessment to guide individualized management. Age-specific hemodynamic profiles highlight the need for standardized diagnostics, prospective validation of monitoring tools, and phenotype-guided interventions to improve outcomes in both adult and pediatric sepsis.

## 1. Introduction

Sepsis is a complex, infection-driven inflammatory process that triggers cascades of interdependent multi-organ failure with life-threatening consequences. Septic shock represents the clinical manifestation of severe septic cardiovascular instability, tissue hypoperfusion, and increased metabolic demands, associated with inadequate oxygen delivery or impaired mitochondrial oxygen utilization [1,2]. Myocardial dysfunction is a frequent complication of sepsis, with diverse clinical manifestations in both pediatric and adult patients [3].

Sepsis-induced cardiomyopathy (SCM) is generally described as an acute, non-ischemic, and potentially reversible form of myocardial dysfunction related to sepsis, ranging from subclinical biventricular impairment to overt cardiogenic shock [4,5,6]. Current definitions of SCM typically include reversible myocardial dysfunction, relative unresponsiveness to fluid resuscitation or vasoactive therapies, and the essential exclusion of acute ischemic coronary syndromes [7]. Importantly, SCM should not be regarded as synonymous with cardiogenic shock. SCM represents a dynamic and heterogeneous spectrum of myocardial dysfunction that may occur across a wide range of hemodynamic states, including preserved or even elevated cardiac output in the setting of profound vasoplegia. From a clinical perspective, septic shock is commonly characterized by vasoplegia, reduced systemic vascular resistance, and frequently hyperdynamic cardiac function, with treatment strategies primarily aimed at restoring vascular tone. In contrast, SCM encompasses a spectrum of myocardial dysfunction, from subtle impairment with typical features of septic shock to severe pump failure resembling cardiogenic shock. In the severe cardiogenic shock setting, SCM patients present with inadequate cardiac output, elevated ventricular filling pressures, increased systemic vascular resistance, tissue hypoperfusion, and evidence of end-organ damage despite optimized preload and afterload [8,9].

Myocardial involvement should be suspected in septic patients with refractory shock, low mixed venous oxygen saturation (SvO_2_), a history of underlying heart disease, or elevated cardiac biomarkers [9]. Reported risk factors for SCM include younger age, pre-existing cardiac disease, higher lactate levels, and positive blood cultures, although their relative contribution remains incompletely defined [10,11].

Myocardial and circulatory compromise in sepsis is closely linked to systemic inflammation, altered blood flow distribution, and cellular metabolic dysfunction. Hemodynamic instability in this context refers to alterations in cardiac output, vascular tone, and tissue perfusion that may result in organ dysfunction. Direct myocardial depression, circulatory alterations, and mitochondrial dysregulation are increasingly recognized as interconnected contributors to SCM [12]. Nevertheless, clinical markers of septic shock and septic cardiomyopathy remain elusive and often demonstrate limited sensitivity and specificity. This limitation is particularly relevant in pediatric patients, with hypotension frequently representing a late sign of shock, and surrogate markers of tissue perfusion, such as serum lactate, mixed venous oxygen saturation (SvO_2_) and base deficit, may provide more reliable assessments [2].

Early fluid resuscitation remains a cornerstone of septic shock management, aiming to increase venous return, cardiac output, and oxygen delivery (DO_2_) to tissues. However, monitoring the deleterious effects of excessive resuscitation is equally essential to prevent fluid overload [13]. Accumulating evidence suggests that only 50–60% of patients are fluid responsive during the early stages of sepsis, while excessive fluid administration may contribute to adverse outcomes [14,15]. Consequently, detailed and continuous hemodynamic assessment is of paramount importance to guide goal-directed therapy in septic patients, as isolated echocardiographic “snapshots” may not fully capture the dynamic nature of cardiovascular dysfunction [16]. Over recent decades, clinical and research focus has shifted toward multimodal monitoring strategies and individualized, physiology-based management approaches in both adult and pediatric sepsis, rather than relying solely on standard clinical parameters or strictly protocolized care [2].

Despite growing recognition of SCM, significant gaps remain regarding its standardized definition, reliable diagnostic criteria, and the optimal integration of hemodynamic monitoring into clinical decision-making, particularly in pediatric populations. Given the profound developmental differences, pediatric septic cardiomyopathy represents a distinct clinical entity rather than a simple adaptation of adult disease. Therefore, the present review aims to provide a comprehensive narrative synthesis of current evidence on SCM, emphasizing differences between adult and pediatric presentations, limitations of current diagnostic and monitoring tools, and existing evidence gaps, with the objective of highlighting priorities for future research on SCM.

## 2. Methods

### 2.1. Study Design

This manuscript is a narrative review with a focused literature search, aiming to synthesize current evidence on septic cardiomyopathy in adult and pediatric patients, with particular emphasis on diagnostic challenges, hemodynamic monitoring, and therapeutic implications.

### 2.2. Search Strategy

A structured literature search was performed primarily in PubMed/MEDLINE. The search included articles published from January 2000 to December 2025. The following keywords were used in various combinations: “pediatric sepsis”, “adult sepsis”, “septic shock”, “septic cardiomyopathy”, “sepsis-induced myocardial dysfunction”, “hemodynamic monitoring”, “echocardiography”, and “transpulmonary thermodilution”. An illustrative example of a search combination is: (“pediatric sepsis”) AND (“septic cardiomyopathy”) AND (“hemodynamic monitoring”). Searches were performed iteratively, and additional relevant studies were identified through manual screening of reference lists of included articles.

### 2.3. Eligibility Criteria

**Figure 1:**
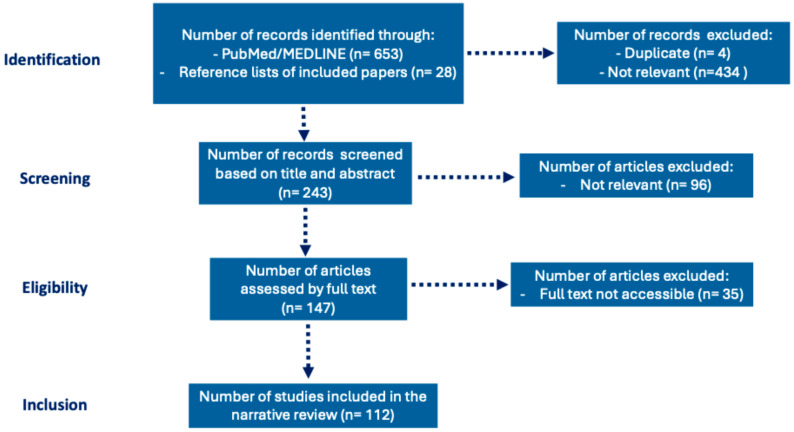
Illustrative study selection flow chart (PRISMA-like) for this narrative review.

All original research, observational studies, meta-analyses, and relevant review articles were included if they focused on: (1) pediatric or adult septic myocardial dysfunction, (2) current evidence on diagnostic and hemodynamic monitoring, and (3) therapeutic strategies in adult and pediatric sepsis. Studies were excluded if they were case reports, non-English publications, animal-only studies, lacked hemodynamic data, or focused on non-septic cardiac dysfunction. The study selection process is illustrated in a PRISMA-like flow diagram, reflecting the narrative review approach without implying a rigid systematic selection (Figure 1).

### 2.4. Data Synthesis

Given the heterogeneity of current studies, a qualitative synthesis of existing evidence on SCM was performed. Adult and pediatric studies were analyzed separately and compared in a narrative way. The strength and limitations of each study were critically assessed, including study design, sample size, and consistency of findings. Formal systematic quality assessment or grading of evidence was not applied, but a descriptive appraisal highlighted the predominance of observational data and the limited availability of high-quality studies on this challenging topic, particularly in pediatric patients.

## 3. Epidemiology

Reported epidemiological estimates of SCM vary widely across studies, reflecting differences in patient populations, diagnostic criteria and monitoring techniques [12,17]. Consequently, prevalence and outcome data should be interpreted with caution, especially when comparing adult and pediatric populations. Age-specific clinical and developmental norms, comorbidities, and prognostic outcomes further differentiate pediatric septic sequelae from adult responses [18]. Both children and adults exhibit septic myocardial dysfunction at similar rates, but with variable clinical manifestations [19]. Recent studies have shown that sepsis accounts for approximately one-quarter of global deaths and nearly 7% of all pediatric deaths, with cardiovascular dysfunction being a frequent complication in both age groups [20,21]. Mortality rates for septic shock range from 40 to 80% in global reports [22], while hospital mortality has been reported to be higher in septic myocardial dysfunction compared to non-septic cardiomyopathies [23].

Prevalence estimates of SCM in adult intensive care units range widely, up to 70%, depending on study design and diagnostic criteria [1,24,25]. A recent meta-analysis estimated a pooled SCM prevalence of approximately 20%, with an associated odds ratio for mortality of 2.3 (95% CI: 1.43–3.69) [19]. Functional cardiac abnormalities in SCM are heterogeneous. In the acute phase, approximately 30–50% of patients may exhibit a hypodynamic or even hyperdynamic myocardial contractility response with preserved cardiac output (CO) [26]. In particular, left ventricular (LV) systolic and diastolic dysfunction have been reported in 50–60% of adult patients with sepsis [12,27,28]. Right ventricular (RV) dysfunction complicates the clinical course in a significant subset of SCM cases (frequently >50%) [29]. Both LV diastolic dysfunction and RV dysfunction have been associated with adverse outcomes [12,27,30,31]. Consistent with these findings, a recent study reported that 78% of adult patients exhibited elevated ejection fraction (EF) with low afterload, while only 22% of cases presented with low EF [32]. ICU mortality in adult SCM is notably high (40–70%), underscoring its potential clinical relevance in sepsis [27].

### Pediatric Prevalence of Septic Cardiomyopathy

In pediatric sepsis, reported SCM prevalence ranges from 30% to 70% [3,16,21]. Studies indicate that 32–47% of children present with systolic dysfunction, while 25–31.5% of cases demonstrate severe biventricular dilatation and dysfunction [16,21,33]. While the majority of children present with low or normal cardiac output (CO), a subset exhibits a high CO profile, reflecting heterogeneity in pediatric shock phenotypes [34]. Moreover, a recent study found that pediatric patients with septic shock present with higher cardiac output and cardiac contractility, whereas patients with cardiogenic shock present with higher preload and afterload indices [22]. In children with refractory septic shock, LV dysfunction was observed in 72% of patients, with concurrent RV dysfunction in 63% [30]. Another study reported that 37% of septic children had isolated LV systolic dysfunction, 33% LV diastolic dysfunction, and 17% of cases presented with both LV systolic and diastolic dysfunction at admission [16]. Pediatric SCM may represent a subgroup with myocardial dysfunction that often shows poor response to fluid and inotropic support [7]. Pediatric SCM mortality has been reported as high as 55%, compared with 7.5% in septic children without myocardial dysfunction [21], reflecting trends similar to those observed in adult cohorts, but with age-specific hemodynamic differences.

## 4. Pathophysiological Mechanisms in Sepsis

The pathophysiology of SCM has not yet been completely elucidated. Early hypotheses of myocardial ischemia have largely been abandoned due to mounting evidence of activation of inflammatory and metabolic pathways, as well as preserved coronary blood flow, limited myocardial oxygen utilization, and the reversibility of myocardial injury [35,36]. At the cellular level, impaired myocardial calcium responsiveness and dysregulated intramyocyte calcium homeostasis, oxidative and nitrosative stress, and mitochondrial dysfunction contribute to impaired myocardial energetics and contractility [7,37]. Given the high mitochondrial density of cardiomyocytes [25], mitochondrial inhibition has been proposed to represent a protective, adaptive bioenergetic response, analogous to myocardial hibernation rather than irreversible injury [38].

Inflammatory signaling pathways, including activation of the NLRP3 inflammasome and caspase-1 mediated pyroptosis in macrophages, cardiomyocytes, and endothelial cells, further depress myocardial function [39,40]. These mechanisms are frequently associated with reversible dysfunction, characterized by global myocardial edema and inflammation without fibrosis, and supported by reversible cardiac magnetic resonance imaging (MRI) findings, which differentiate SCM from myocardial ischemia or acute myocarditis [41]. Beyond direct myocardial effects, vasoplegia induced by nitrosative stress, autonomic dysfunction due to sympathetic overstimulation, β-adrenergic receptor desensitization, along with microcirculatory dysfunction with capillary leakage, endothelial dysfunction, and procoagulant phenomena may further compromise myocardial responsiveness to endogenous and exogenous catecholamines, contributing to the multifactorial pathophysiology of SCM [37,42].

These pathophysiological mechanisms have important clinical implications, as the complexity of myocardial dysfunction in sepsis may be further complicated by profound autonomic dysregulation [43], resulting in sympathetic overactivation, tachycardia, increased myocardial oxygen demand, and impaired diastolic perfusion. Autonomic imbalance also modulates immune responses, potentially promoting immunosuppression [44]. Based on this concept, stress-induced myocardial dysfunction, classically described as Takotsubo syndrome, shares overlapping pathophysiological features with SCM, including catecholamine excess, microvascular dysfunction, and myocardial stunning. Cardiac magnetic resonance imaging further supports this overlap by demonstrating myocardial edema, consistent with a reversible, non-ischemic, stress-related pattern. Collectively, these observations suggest that SCM may represent part of a broader spectrum of stress-induced myocardial dysfunction rather than a distinct entity, with relevant diagnostic and therapeutic implications [41].

### Pediatric Pathophysiological Responses

While the fundamental mechanisms of SCM appear broadly similar across ages, developmental differences substantially shape pediatric phenotypes [45]. Children have lower myocardial reserve, immature autonomic regulation of vascular tone, and greater sensitivity to afterload changes, making them more prone to low cardiac output and elevated afterload (“cold shock” states), in contrast to the hyperdynamic vasoplegic phenotype commonly observed in adults [3,16,45,46].

In addition, pediatric cardiomyocytes may also exhibit immature, age-dependent differences in β-adrenergic receptor density and signaling, including incomplete receptor-G-protein coupling, resulting in blunted inotropic responses to β-agonists [47,48]. Mitochondrial immaturity may further compromise contractile function and delay functional recovery following the septic insult [49]. Importantly, most pediatric mechanistic data are extrapolated from adult studies, highlighting a substantial knowledge gap in children [50,51].

Collectively, these interconnected mechanisms may form the biological basis for the heterogeneous echocardiographic and hemodynamic phenotypes observed in septic cardiomyopathy.

## 5. Echocardiographic Findings in Sepsis

Transthoracic echocardiography represents a cornerstone of bedside cardiovascular assessment in septic patients and plays a central role in characterizing adult and pediatric myocardial dysfunction [52,53]. Echocardiography allows rapid evaluation of variable abnormalities in septic patients, enabling clinicians to identify the type of cardiogenic dysfunction (left, right, biventricular, regional, or apical wall motion abnormalities, e.g., in Takotsubo cardiomyopathy) [8]. Moreover, echocardiography allows rapid evaluation of the underlying cause of low cardiac output (CO) and facilitates identification of eligible patients for mechanical circulatory support [54,55]. Left ventricular ejection fraction (LVEF) is a commonly used indicator of myocardial contractility, but its afterload dependence limits its accuracy [56]. On the contrary, an extensive body of literature focuses on global longitudinal strain (GLS) as a more sensitive and specific parameter for SCM, and as a significant predictor of higher mortality [57,58].

Biventricular dysfunction appears to be relatively common in SCM [59]. Overall, most adult patients present with global left ventricular (LV) hypokinesia upon admission, or this finding might be unmasked after vasopressor support initiation, possibly reflecting the influences of loading conditions [60]. One commonly reported feature of SCM is LV diastolic dysfunction accompanied by LV dilatation and increased LV end-diastolic volume (LVEDV), often occurring despite low or normal filling pressures, which may act as a compensatory mechanism to preserve stroke volume [61]. RV dysfunction is another critical component in SCM, induced by multiple factors, including mechanical ventilation, hypoxemia, and hypercapnia [8,9,26,62]. It seems that the combination of decreased preload, increased RV afterload, and compromised RV contractility likely explains why the RV cannot keep pace with the LV, despite ventricular interdependence in steady states [63].

Interestingly, there are multiple reports, especially in adults, supporting that a low LV ejection fraction (EF) combined with LV dilatation tends to correlate with a “paradoxical” lower mortality and better outcomes [64,65]. This paradox might be explained by the fact that vasoplegia and distributive shock (warm shock phenotype) may often pseudo-normalize a low EF and mask cardiac impairment, enabling a severely dysfunctional heart to pump a seemingly normal cardiac output, while less severe patients without profound shock tend to reveal their actual low EF [9,43]. Clinicians should also keep in mind that LV dysfunction may occur due to vasopressor-driven afterload increase, with dynamic obstruction by inotropic or vasopressor agents. It is also essential to carefully assess CO, as inotropic medications are only indicated if the cardiogenic shock is associated with low CO and hypoperfusion [54]. Echocardiography studies can roughly estimate cardiac output using LV outflow tract velocity integral (LVOT VTI). Selected adult cohorts have reported normal LVOT VTI values of >18 cm, while observational studies in patients with SCM typically report lower LVOT VTI, and a cutoff of less than 13.2 cm has been associated with a higher risk of mortality [66,67].

### Pediatric Echocardiography

Pediatric SCM exhibits distinct echocardiographic patterns due to developmental physiology and shock phenotypes, with a high prevalence of LV dysfunction [16,46,68]. Infants and young children with septic shock often present with increased afterload and low cardiac output (“cold shock”) [69,70]. Multiple studies have also shown that serum troponin levels correlate with myocardial impairment and disease severity [16,71]. However, the potential direct or indirect interactions between myocardial dysfunction and mechanical ventilation, pulmonary vascular resistance, hypoxemia or medications are not yet fully understood [16].

Conventional echocardiographic parameters evaluating cardiac function, like ejection fraction (EF), fractional shortening (FS), fractional area change (FAC) and tricuspid annular plane systolic excursion (TAPSE), might underestimate the severity of SCM, due to preload and afterload dependence [43,57,68]. This limitation is particularly important for pediatric patients, as low cardiac output may coexist with normal EF, potentially masking myocardial dysfunction [72]. Emerging, objective echocardiographic techniques, including tissue Doppler imaging (TDI) and strain imaging with speckle-tracking echocardiography (STE), may detect early changes in myocardial contractility before abnormalities appear in conventional parameters [7,27,72]. Both LV and RV wall strain have been independently associated with mortality in pediatric SCM, suggesting a potential role for strain imaging in detecting subclinical dysfunction and improving risk stratification [72,73]. Interestingly, conventional echocardiography demonstrated a diagnostic ability of approximately 30% for LV and RV dysfunction, respectively, while speckle tracking echocardiography revealed LV and RV dysfunction in approximately 70% of cases, respectively [74]. Future research may further clarify the diagnostic value of myocardial strain imaging, guiding appropriate therapeutic interventions for SCM. Given the limitations of static echocardiographic parameters, complementary hemodynamic monitoring is often required.

## 6. Basic Hemodynamic Monitoring

Hemodynamic monitoring is a critical component of SCM management, facilitating assessment of cardiac function, intravascular volume status, and response to therapeutic measures [75]. Commonly used basic or “static” hemodynamic parameters include heart rate (HR), blood pressure (BP), urine output, capillary refill time (CRT), serum lactate, central venous pressure (CVP), pulmonary capillary wedge pressure, cardiac output (CO), mixed venous saturation (SvO_2)_ and venous-to-arterial carbon dioxide difference (Pv-aCO_2_). While these variables provide valuable clinical information, they lack specificity when used as isolated modalities to direct therapeutic interventions [2,76]. Despite these limitations, basic hemodynamic parameters remain clinically relevant as early warning signs of hypoperfusion and circulatory collapse rather than definitive diagnostic tools.

SvO_2_ reflects tissue oxygen delivery and oxygen consumption. In septic patients, it is expected to be normal or elevated. A low SvO_2_ (<70%) may identify patients with inadequate cardiac output, prompting consideration of targeted interventions, such as fluid optimization, inotropic support, or blood transfusion. Pv-aCO_2_ serves as a surrogate marker of anaerobic metabolism with faster responses to resuscitation compared to lactate. Pv-aCO_2_ is inversely related to CO, and elevated values (Pv-aCO_2_ > 6 mmHg) may indicate inadequate fluid resuscitation [54,75]. Serial lactate measurements remain widely used and have been associated with myocardial dysfunction and adverse outcomes in sepsis [21]. However, lactate elevation is multifactorial and should be interpreted in conjunction with other hemodynamic and perfusion indices, rather than an isolated marker [77]. Moreover, several circulating biomarkers correlate with myocardial involvement. Elevated B-natriuretic peptide (BNP) is inversely correlated with fractional shortening and directly associated with the severity of sepsis, particularly in pediatric populations, although its diagnostic value in adult sepsis remains inconsistent. Conversely, although cardiac troponins correlate with myocardial injury and mortality in adult sepsis, their prognostic role in pediatric septic shock is less clearly defined [3,78].

Dynamic parameters of fluid responsiveness, such as passive leg raising (PLR), variations in inferior vena cava (IVC) diameter, stroke volume variation (SVV), and pulse pressure variation (PPV), offer improved assessment of preload responsiveness [79]. However, their applicability in pediatric patients remains limited due to age- and ventilation-related factors [2]. Even though specific goals in terms of macrohemodynamics are often easily achieved in sepsis, occult microcirculatory failure might be imminent, representing a potential determinant of septic shock evolution [20]. Therefore, evolving parameters of microcirculatory perfusion (sublingual microcirculatory alterations, tissue red blood cell perfusion) are currently analyzed for their potential clinically relevant benefits in patient outcomes [13]. Microcirculatory monitoring remains largely investigational, especially in children, with evidence suggesting that microcirculatory alterations may precede overt hypotension or low cardiac output [80,81]. Table 1 highlights variations in echocardiographic and hemodynamic findings between adult and pediatric SCM, illustrating how developmental physiology influences monitoring interpretation. Integrating multiple parameters is essential to interpret macro- and microcirculatory patient status, linking basic monitoring with advanced techniques discussed next.

## 7. Advanced Multimodal Hemodynamic Monitoring

Advanced monitoring techniques aim to provide continuous, objective and integrative assessment of cardiovascular performance, guiding individualized therapeutic strategies. These techniques are increasingly evaluated in both adult and pediatric populations, although pediatric-specific normative values remain limited. Invasive, minimally invasive, and non-invasive techniques offer complementary advantages, with differences in accuracy, trend monitoring, and procedural requirements that should be considered in both clinical decision-making and resource-limited contexts [20,75,83].

Minimally invasive monitoring may enable the definition of hemodynamic phenotypes with objective and dynamic parameters [37,84,85]. Transpulmonary thermodilution (TPTD) devices, such as pulse index continuous cardiac output (PiCCO) [86], can provide information regarding CO, stroke volume, afterload parameters, volumetric preload indices (global end-diastolic volume/GEDV), and pulmonary edema severity, through extravascular lung water (EVLW) and vascular permeability indicators [22,54]. A recent pediatric study demonstrated that decreased afterload, as assessed by systemic vascular resistance (SVR) derived from TPTD, was significantly associated with mortality in septic shock, whereas cardiac index (CI) predicted mortality only in cardiogenic shock phenotypes [22]. Compared with echocardiography, TPTD provides the advantage of continuous trend monitoring, facilitating real-time evaluation of hemodynamic responses to therapy, but requires specialized equipment and trained personnel, limiting its feasibility in low-resource settings [87].

In adults, TPTD-derived CO measurements show reasonable agreement with echocardiographic estimates [88], although substantial interindividual variability exists, suggesting that both methods provide complementary information rather than serving as interchangeable modalities [85,89]. Interpretation of advanced hemodynamic data in pediatric patients remains particularly challenging due to limited age-specific normative values and developmental physiological differences [90].

Regarding pulmonary edema estimation, adult studies have shown a strong correlation between elevated EVLW and pulmonary fluid, whereas this relationship has not been fully confirmed in children [91]. Similarly, adult reference ranges for GEDV and EVLW cannot be directly applied to younger children, who often exhibit lower GEDV and higher EVLW values [92].

Pulmonary artery catheterization (PAC) provides comprehensive hemodynamic data but has not demonstrated a clear survival benefit in adults and carries procedural risks, limiting its use, particularly in children [8,54]. Therefore, choice of monitoring modality should balance precision with safety, feasibility, and availability, especially in resource-constrained environments [93]. Minimally invasive techniques such as TPTD may represent a feasible alternative in pediatric patients, showing reasonable agreement with PAC, and the ability to detect clinically relevant changes in CO of approximately 12% [85,91].

Several important limitations of TPTD, primarily described in adult populations but likely relevant to children, must be acknowledged. The technique does not directly measure myocardial contractility or ventricular filling pressures, and its accuracy may be compromised in the presence of intracardiac shunts, due to recirculation of the infused ice-cold saline [85,94]. As previously noted, a normal TPTD CO does not rule out SCM, especially in patients with low SVR, emphasizing that cardiac output should be interpreted in conjunction with afterload [43]. Arrhythmias, inadequate sedation, changes in ventilatory settings, and tricuspid regurgitation may also affect CO reliability [89].

From a feasibility perspective, basic clinical parameters and bedside echocardiography remain the cornerstone of hemodynamic assessment, particularly in clinical settings with limited access to advanced monitoring technologies. Echocardiography provides a rapid, non-invasive method to assess cardiac function and CO trends, and is often the only practical tool in resource-limited environments. Integration with basic parameters allows clinicians to make informed therapeutic decisions without reliance on advanced devices [95].

Importantly, advanced monitoring alone is unlikely to improve outcomes unless it is embedded within a pathophysiology-driven and clinically integrated decision-making framework [96]. Table 2 provides a structured overview of diagnostic and hemodynamic monitoring tools used in SCM, highlighting their applicability in pediatric patients.

## 8. Current Treatment Concepts Based on Hemodynamic Monitoring

Given the marked heterogeneity of SCM, no single therapeutic strategy can be universally recommended. Therapeutic decisions should therefore be individualized based on the predominant hemodynamic phenotype as defined by integrated clinical, echocardiographic, and hemodynamic monitoring, and its dynamic evolution over time [98,99].

In patients with preserved cardiac output and vasoplegia, vasopressor therapy aimed at restoring vascular tone is supported by current sepsis guidelines rather than SCM-specific trials. Early initiation of norepinephrine has been shown to improve mean arterial pressure (MAP), venous return, and cardiac output, with indirect evidence suggesting improved microcirculatory perfusion, and it remains the first-line agent in both adult and pediatric septic shock [100,101]. In contrast, patients with impaired myocardial contractility and low cardiac output may benefit from cautious inotropic support after optimization of preload and afterload. Dobutamine is commonly used as a first-line inotropic agent due to its easily titratable and predictable hemodynamic effects, although available studies do not demonstrate clear superiority over other inodilators, nor consistent outcome benefits [102,103]. Evidence supporting improved outcomes with inotropic therapy in SCM remains limited, particularly in children, and inappropriate use may increase myocardial oxygen demand, provoke arrhythmias, or worsen hypotension [104,105]. Accordingly, initiation and titration of vasopressors and inotropes should be guided by repeated integration of both basic and advanced hemodynamic data. Importantly, different clinical phenotypes may evolve dynamically over the course of sepsis, especially in pediatric patients, highlighting the need for serial hemodynamic evaluations [8,9,54].

Vasopressin may be considered a second-line treatment and an adjunct to norepinephrine in refractory vasoplegia, aiming at further increasing MAP while sparing an additional adrenergic burden. Its use is largely extrapolated from adult data but has not been specifically validated in SCM, and caution is recommended in patients with impaired cardiac output due to potential reductions in coronary perfusion [106,107,108].

There are no clear recommendations on other medications aiming at improving hemodynamics, like angiotensin II or methylene blue, which have been proposed for refractory vasoplegia, although their role in SCM has not been systematically evaluated [109,110,111]. Emerging interest may also focus on adrenergic modulation agents, like α2-agonists (e.g., dexmedetomidine), due to their potential anti-inflammatory effects, beneficial microcirculatory actions, reduction in sympathetic overactivation, and vasopressor-sparing properties. In the same context, short-acting β1-selective blockers, such as esmolol or landiolol, have been investigated in adult SCM, with studies suggesting potential benefits in controlling heart rate, reducing catecholamine-driven cardiotoxicity, myocardial oxygen demand, and improving diastolic filling. However, these findings are largely derived from small adult studies and may not be generalizable, while these agents may be harmful in the presence of ongoing hypoperfusion [112]. Pediatric evidence for these supportive agents is currently limited to experimental studies or small clinical cohorts [13]. Consequently, while these therapies show mechanistic promise and may be considered on a case-by-case basis in experienced centers with continuous hemodynamic monitoring, routine use cannot currently be recommended, particularly in children. Overall, most therapeutic recommendations in SCM are guided by physiological rationale, observational studies or expert consensus, rather than high-quality randomized evidence, highlighting the need for cautious, individualized bedside interpretation, particularly in pediatric patients [13,54].

### 8.1. Refractory Septic Shock

Refractory septic shock is commonly defined in adult and pediatric populations as persistent circulatory failure with severe hyperlactatemia (serum lactate > 8 mmol/L) and a high vasoactive inotrope score (VIS; thresholds vary in adults, with >20 commonly used in pediatric literature), despite adequate vasoactive support [113,114]. These thresholds are primarily derived from pediatric observational studies and expert consensus, and definitions vary considerably across studies, limiting their universal applicability [115,116,117]. Notably, refractory shock may occur despite apparently preserved macrohemodynamic indices, such as normal SvO_2_ and Pv-aCO_2_, suggesting that microcirculatory and mitochondrial dysfunction may predominate and contribute to poor responsiveness to initial therapeutic interventions [75].

Potentially reversible causes and underlying cardiac pathologies, including myocarditis, myocardial infarction, cardiomyopathy, or congenital heart disease, should initially be systematically excluded. Echocardiography plays a central role in this evaluation and should be repeatedly performed in refractory shock to assess cardiac function, loading conditions, and to identify complications related to high-dose catecholamine therapy, such as LV outflow tract obstruction [13].

In selected patients with refractory septic shock, invasive mechanical circulatory support, including ventricular assist devices (VADs) or venous-arterial extracorporeal membrane oxygenation (VA-ECMO), may be considered rescue or bridge therapies [118,119,120]. Observational data suggest that these modalities can be lifesaving in pediatric and adult patients, although outcomes depend on patient selection, timing of initiation, and the reversibility of the underlying disease process. Given the high resource utilization and potential complications associated with mechanical support, multidisciplinary evaluation and integration of advanced hemodynamic monitoring are essential when considering these interventions, particularly in children [121,122,123].

### 8.2. Post-Sepsis Cardiac Recovery

Emerging evidence indicates that, although SCM has classically been described as a transient and reversible condition, recent cardiac magnetic resonance imaging studies suggest that a substantial proportion of survivors exhibit persistent structural and functional abnormalities, including LV dilatation, reduced systolic function, and non-ischemic myocardial fibrosis after recovery. This persistent phenotype, often referred to as post-sepsis cardiomyopathy, has important long-term implications for myocardial function, heart failure risk, and sustained therapeutic interventions [28]. Pediatric data remain sparse, although available studies suggest generally favorable cardiac recovery, with incomplete resolution in a minority of patients [124].

## 9. Discussion

Septic cardiomyopathy (SCM) is a complex, heterogeneous and frequently underdiagnosed manifestation of sepsis, affecting both adult and pediatric populations [62]. Despite increasing recognition, the true burden of SCM remains difficult to define due to variable diagnostic strategies and age-dependent physiological differences [98]. Pediatric epidemiological data are often derived from small, single-center studies, which limits generalizability, highlighting a key evidence gap in defining pediatric SCM prevalence and outcomes [21,33]. Observed variability in reported prevalence and outcomes likely reflects differences in study design, timing of assessment, illness severity, and diagnostic criteria, underlying the complexity of sepsis phenotyping and treatment approaches [9]. The clinical impact of SCM cannot be conceptualized as an isolated cardiac phenomenon, but rather arises from the integration of systemic inflammation, vasoplegia, autonomic dysregulation, microcirculatory failure and mitochondrial energetic impairment [4,37,49]. This integration highlights the need for clinicians to interpret observed echocardiographic and hemodynamic findings within the broader systemic context [98].

Overall, pediatric SCM reflects age-specific hemodynamic vulnerabilities superimposed on common pathophysiologic mechanisms. Pediatric patients may present with low cardiac output and high afterload (“cold shock”), whereas adults more often display hyperdynamic vasoplegia. Recognizing SCM physiology patterns is critical, as developmental differences in autonomic regulation, myocardial reserve, and β-adrenergic signaling in children may amplify the clinical impact of myocardial depression and influence the response to fluids, vasoactive agents, and inotropes [48,125]. These age-specific features highlight the need for phenotype-guided monitoring and therapy. The multifactorial pathophysiology possibly explains the wide variability in reported prevalence, phenotypes and outcomes across studies, leaving uncertainty about which parameters or interventions significantly affect prognosis [19,45,47].

Traditional echocardiographic parameters, such as left ventricular ejection fraction (LVEF), are highly dependent on preload and afterload, possibly underestimating myocardial impairment [4,32,68]. This limitation may partially explain the paradoxical association between reduced LVEF with ventricular dilatation and improved outcomes in some adult studies, potentially reflecting preserved preload reserve and adaptive myocardial responses rather than irreversible myocardial failure [21,64,126]. These opposite trends likely reflect methodological heterogeneity and differences in loading conditions at the time of assessment [4]. Consequently, reliance on isolated static echocardiographic measurements is insufficient for accurate characterization of SCM [43]. Emerging echocardiographic techniques, including tissue Doppler imaging and speckle-tracking-derived myocardial strain, may be more sensitive in detecting early and subtle myocardial abnormalities [4,7,56], and provide better prognostic discrimination [73,74]. However, their clinical integration remains complementary, due to operator variability and lack of standardized reference values, particularly in pediatric populations [7,16].

Hemodynamic monitoring constitutes another cornerstone of SCM assessment and management. Static macrohemodynamic parameters, such as heart rate, blood pressure, central venous pressure, or even cardiac output, often fail to reflect tissue perfusion and myocardial performance in sepsis [75,97]. Advanced and multimodal monitoring may allow clinicians to relate observed hemodynamic patterns to underlying mechanisms, guiding physiologically appropriate interventions [90,127,128] and generally showing reasonable agreement with echocardiography [22,88,89]. However, as emphasized above, advanced monitoring does not always lead to improved outcomes, unless embedded within a personalized, pathophysiology-guided clinical context [13,54].

**Figure 2:**
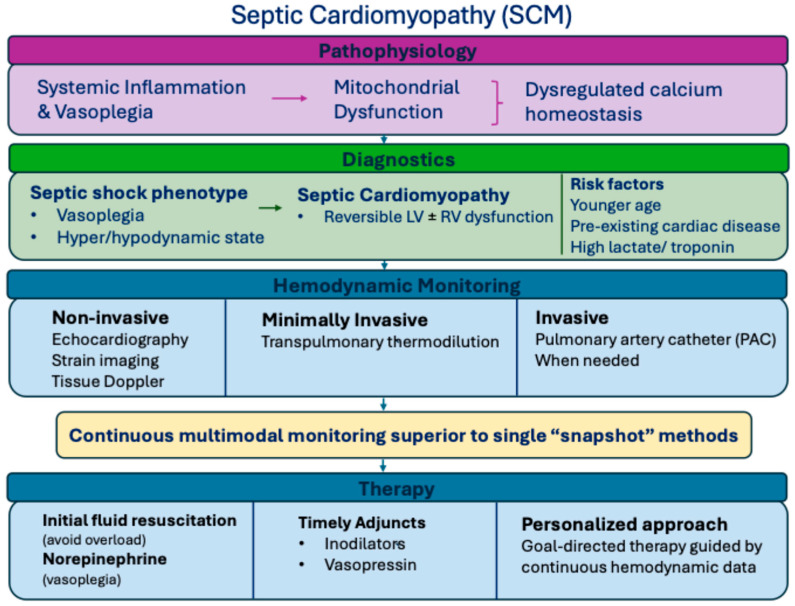
Integrated overview of septic cardiomyopathy, serving as a conceptual framework. Systemic inflammation triggers myocardial dysfunction, requiring careful diagnostic steps, multimodal hemodynamic monitoring, and tiered therapeutic strategies.

Evidence on the dynamic and potentially reversible nature of SCM [129] highlights the need for repeated and longitudinal assessments, particularly in pediatric patients [21,50,128]. Clinical decisions should therefore be individualized and responsive to dynamic patient status, with continuous evaluation of cardiac function, systemic vascular resistance and tissue perfusion, recognizing that a “normal” cardiac output may mask significant myocardial dysfunction in vasoplegic states [9,43,54]. Taken together, these considerations illustrate the need to integrate mechanistic insights with bedside evaluation, tailoring fluid, vasoactive, and inotropic therapy to patient-specific hemodynamic and developmental profiles, as illustrated in Figure 2.

Pediatric SCM is characterized by earlier reliance on echocardiography rather than invasive hemodynamic monitoring, age-specific hemodynamic patterns, and the absence of validated diagnostic thresholds for systolic or diastolic dysfunction. Pediatric monitoring protocols are therefore largely individualized, with greater emphasis on serial assessment rather than standardized targets [128]. Outcomes in children appear more heterogeneous, with generally favorable recovery in survivors but limited data on long-term myocardial sequelae. Together, these differences highlight pediatric SCM as a distinct clinical and research entity, requiring age-specific diagnostic frameworks, monitoring strategies, and outcome measures. Accordingly, pediatric disease should not be viewed merely as an extension of adult disease, but rather as a condition shaped by developmental cardiovascular physiology, distinct shock phenotypes, and substantial diagnostic uncertainty [21]. Prospective data confirming outcome benefits of advanced monitoring in pediatric SCM are limited, marking a significant knowledge gap. In practice, SCM management remains largely empirical. Fluid resuscitation, vasoactive medications, and inotropes are guided by physiologic rationale rather than robust evidence, while uniform resuscitation targets may be harmful in certain phenotypes [15,100].

These limitations highlight two critical gaps: (1) the absence of standardized, age-appropriate diagnostic criteria for SCM, and (2) the lack of prospective, interventional studies evaluating whether phenotype-specific monitoring or hemodynamic-guided therapy can improve outcomes. Moreover, most studies focus on macrohemodynamic parameters, whereas microcirculatory and mitochondrial dysfunction likely contribute to refractory shock, necessitating novel measurement techniques [49,130]. Future research should integrate mechanistic insights into study design to link underlying pathophysiology with observed clinical and hemodynamic responses.

## 10. Limitations

This review has several important limitations that must be acknowledged when interpreting its findings. Although a structured literature search was performed, this work represents a narrative rather than a systematic review, and therefore selection bias cannot be excluded. Additionally, the available literature on septic cardiomyopathy is characterized by substantial heterogeneity in study design, patient populations, and diagnostic criteria. Substantial knowledge gaps remain, despite increasing recognition of SCM, especially in pediatric populations, regarding standardized definitions and protocols, limiting comparability between studies and reducing definitive conclusions. Formal assessment of risk of bias or grading of evidence quality or strength of recommendations was not performed, and conclusions of this review should therefore be interpreted as descriptive and hypothesis-generating rather than definitive.

The evidence base underlying SCM is predominantly derived from observational studies, small cohorts, and retrospective analyses. As a result, the overall strength of evidence supporting epidemiologic estimates, mechanistic pathways, and therapeutic strategies remains moderate to low. Selection bias, survivor bias, and confounding by illness severity are common limitations, particularly in studies relying on echocardiographic assessment performed at variable time points during resuscitation. Importantly, pediatric evidence remains limited, with most data derived from small, observational, or single-center studies. Consequently, many mechanistic insights, diagnostic thresholds, and therapeutic concepts applied to children are mostly extrapolated from adult studies, despite clear developmental differences in cardiovascular physiology. This limitation highlights the need for standardized definitions and prospective pediatric studies.

## 11. Conclusions

Septic cardiomyopathy often complicates the course of sepsis and is characterized by a dynamic and heterogeneous spectrum of myocardial dysfunction affecting both adults and pediatric patients. Establishing unified consensus and diagnostic criteria for septic cardiomyopathy remains challenging due to the dynamic interplay between intrinsic myocardial depression and sepsis-related alterations in preload, afterload, and vascular tone. Current diagnostic approaches are limited. Conventional echocardiographic parameters may be influenced by loading conditions and underestimate myocardial dysfunction, whereas emerging myocardial strain indices and multimodal advanced hemodynamic monitoring provide more sensitive assessment of circulatory phenotypes. These non-invasive and minimally invasive techniques may provide complementary information, facilitating phenotypic characterization and improving diagnostic precision.

Overall, septic cardiomyopathy may be conceptualized as a time-dependent, phenotype-driven condition requiring repeated, integrated cardiovascular assessment rather than reliance on isolated static parameters or protocolized resuscitation. This review highlights key knowledge gaps, including the lack of standardized diagnostic criteria, insufficient pediatric-specific monitoring thresholds, and limited prospective evidence on phenotype-guided interventions. Future research should prioritize multicenter, prospective studies to establish age-appropriate definitions, validate advanced diagnostic tools, and evaluate whether individualized monitoring and therapy improve outcomes in both adult and pediatric SCM.

## Figures and Tables

**Table 1:** Comparison of Adult vs. Pediatric Septic Cardiomyopathy characteristics.

Feature	Adults	Pediatrics
**Predominant shock phenotype**	Hyperdynamic, vasoplegic (“warm shock”) ^1^	Low cardiac output, elevated afterload (“cold shock”) ^2^
**Cardiac output pattern**	Often preserved or high ^1^	Frequently low; some hyperdynamic cases ^2^
**Systemic vascular resistance (SVR)**	Low ^1^	Often elevated; highly sensitive to afterload ^2^
**Myocardial reserve**	Relatively preserved ^1^	Lower; limited ability to respond to stress ^2^
**Echocardiographic findings**	LV diastolic dysfunction common; RV dysfunction in ~30–50% ^1^	LV systolic dysfunction dominant; RV dysfunction in ~60% of severe cases ^2^
**Response to fluids**	Many respond in early phase ^1^	Often poor response; refractory shock common ^2^
**Response to inotropes**	Typically preserved ^1^	Blunted due to immature β-adrenergic signaling ^2^
**Mortality impact**	SCM increases ICU mortality (40–70%) ^3^	SCM dramatically increases mortality (~55% vs. 7.5% without SCM) ^4^

This table summarizes key clinical, hemodynamic, and echocardiographic features of SCM in adults and children. Data should be interpreted in the context of study heterogeneity, methodological limitations, and age-dependent physiological differences. References: ^1^ [1,15]; ^2^ [2,16,82]; ^3^ [1,3,43]; ^4^ [4,30,50]. LV, left ventricle; RV, right ventricle; SCM, septic cardiomyopathy; ICU, intensive care unit.

**Table 2:** Diagnostic and hemodynamic monitoring tools in SCM.

Modality	Parameters Assessed	Clinical Role in SCM	Advantages	Limitations	Pediatric Considerations
**Basic monitoring**	HR, BP, urine output, lactate, SvO_2_, Pv-aCO_2_^1^	Early detection of hypoperfusion	Widely available, non-invasive	Low specificity; isolated markers may mislead ^1^	Hypotension often late; lactate/SvO_2_ may be more reliable ^1^
**Echocardiography**	LVEF, LVEDV, FS, FAC, TAPSE, GLS, RV strain ^2^	Characterize myocardial dysfunction, guide therapy	Bedside, non-invasive, assesses both ventricles	Load-dependent; operator variability ^2^	Age-specific norms needed; strain may detect subclinical dysfunction ^2^
**Dynamic preload indices**	PLR, IVC diameter, SVV, PPV ^2^	Predict fluid responsiveness	Non-invasive or minimally invasive	Limited in spontaneously breathing children; ventilation-dependent ^2^	Pediatric norms not fully established ^2^
**Transpulmonary thermodilution (PiCCO)**	CI, SVR, GEDV, EVLW ^3^	Continuous trend analysis; guide fluids/vasopressors	Measures preload, afterload, pulmonary edema	Invasive; affected by shunts, arrhythmias, ventilator changes ^3^	Reference ranges limited; interpretation must consider low SVR ^3^
**Pulmonary artery catheter**	CO, PA pressures, PCWP ^3^	Detailed hemodynamic assessment	Gold standard for physiology	Procedural risks; no proven survival benefit ^3^	Rarely feasible; invasive in children ^3^

Summary of commonly used modalities in septic cardiomyopathy and reported pediatric considerations. Data should be interpreted in the context of study heterogeneity and methodological limitations, without formal grading of evidence strength. References: ^1^ [2,13,97]; ^2^ [1,2,16,22]; ^3^ [2,3,86,87,88]. HR, heart rate; BP, blood pressure; SvO_2_, mixed venous oxygen saturation; Pv-aCO_2_, venous-to-arterial carbon dioxide difference; LVEF, left ventricle ejection fraction; LVEDV, left ventricular end-diastolic volume; FS, fractional shortening; FAC, fractional area change; TAPSE, tricuspid annular plane systolic excursion; GLS, global longitudinal strain; RV, right ventricle; IVC, inferior vena cava; SVV/PPV, stroke/pulse volume variation; CI, cardiac index; SVR, systemic vascular resistance; GEDV, global end-diastolic volume; EVLW, extravascular lung water.

## Data Availability

No new data were created.

## Abbreviations

The following abbreviations are used in this manuscript:

BP	Blood pressure
CO	Cardiac Output
CRT	Capillary refill time
CVP	Central venous pressure
DO_2_	Oxygen Delivery
EVLW	Extravascular lung water
FAC	Fractional area change
FS	Fractional shortening
GEDV	Global end-diastolic volume
GLS	Global longitudinal strain
HR	Heart rate
ICU	Intensive Care Unit
LVEF	Left ventricular ejection fraction
LVEDV	Left ventricle end-diastolic volume
LV	Left ventricle/ventricular
MAP	Mean arterial pressure
PAC	Pulmonary artery catheter
Pv-aCO_2_	Venous-to-arterial carbon dioxide difference
RV	Right ventricle/ventricular
SCM	Sepsis-induced cardiomyopathy
STE	Speckle-tracking echocardiography
TAPSE	Tricuspid annular plane systolic excursion
TDI	Tissue Doppler imaging
TPTD	Transpulmonary thermodilution
VA-ECMO	Veno-arterial extracorporeal membrane oxygenation
VAD	Ventricular assist device
VIS	Vasoactive inotrope score
SvO_2_	Mixed venous oxygen saturation
PICCO	Pulse index continuous cardiac output
